# Environmentally driven sexual segregation in a marine top predator

**DOI:** 10.1038/s41598-017-02854-2

**Published:** 2017-06-01

**Authors:** Vitor H. Paiva, Justin Pereira, Filipe R. Ceia, Jaime A. Ramos

**Affiliations:** MARE – Marine and Environmental Sciences Centre, Department of Life Sciences, University of Coimbra, 3004-517 Coimbra, Portugal

## Abstract

Sexual segregation in foraging occurs in many animal species, resulting in the partitioning of resources and reduction of competition between males and females, yet the patterns and drivers of such segregation are still poorly understood. We studied the foraging movements (GPS-tracking), habitat use (habitat modelling) and trophic ecology (stable isotope analysis) of female and male Cory’s shearwaters *Calonectris borealis* during the mid chick-rearing period of six consecutive breeding seasons (2010–2015). We found a clear sexual segregation in foraging in years of greater environmental stochasticity, likely years of lower food availability. When food became scarce, females undertook much longer foraging trips, exploited more homogeneous water masses, had a larger isotopic niche, fed on lower trophic level prey and exhibited a lower body condition, when compared to males. Sexual competition for trophic resources may be stronger when environmental conditions are poor. A greater foraging success of one sex may result in differential body condition of pair mates when enduring parental effort, and ultimately, in an increased probability of breeding failure.

## Introduction

Sexual Size Dimorphism (SSD), where one sex is larger than the other, occurs in many wildlife groups such as insects^1^, mammals^2^ or birds^3^. This is found in seabirds, in which sex differences in foraging area (e.g. ref. 3), diving behaviour (e.g. ref. 4), foraging trip duration (e.g. ref. 5) or overall provisioning rate (e.g. ref. 6) are commonly reported. SSD may be related to the different parental roles of each sex. Male-biased SSD is expected to be resultant from sexual selection, as a larger size gives advantage in intra-sexual competition for mates, in attracting females^7^ or territorial defence^8^. Instead, larger females (i.e. reverse sexual dimorphism) can store more energy for reproduction, produce larger eggs, better provision food to their young or defend territories^9^. Furthermore, the Energetic Constraint Hypothesis (ECH) suggests that relative investment by males and females may differ according to breeding stage^10^. For example, the costs incurred by egg production^11^ or unequal contribution to incubation^12^ may translate into females being in poorer condition than males at the onset of the chick-rearing period, and consequently into the need for females to allocate more time to self-provisioning than males. Still, our understanding of how broadly the ECH explains the at-sea behavioural patterns of diverse marine top predators is still limited (but see ref. 13).

SSD in body mass and wing morphology is thought to play a functional role in flight performance and is used to explain differences in the at-sea distribution of male and female seabirds^3^. Partial or complete sexual foraging segregation has been reported in several seabird species, such as black-browed albatrosses *Thalassarche melanophris* ^14^, Cape gannets *Morus capensis* ^15^, Hawaiian petrels *Pterodroma sandwichensis* ^16^ or Scopoli’s shearwaters *Calonectris diomedea* ^17^. However, sexual differences in size and shape may be poor predictors of differentiation in the way male and female seabirds exploit the marine environment, and sexual segregation might be environmentally driven^18^. In some species with SSD, such as Cory’s Shearwater *Calonectris borealis*, recent studies have shown that pronounced sexual size dimorphism in bill size, body mass and wing length does not seem to translate into sexual segregation in foraging distribution, behaviour and trophic ecology, at least during the incubation stage^19, 20^. Therefore, examining other stages of the life cycle of this species is necessary to determine if sexual segregation and sex-specific behaviour are sexually- or environmentally-driven (i.e. through changes in the availability of food resources).

We deployed GPS-loggers on male and female Cory’s shearwaters during August – September of 2010–2015, in order to determine whether a sexual segregation in foraging strategies, at-sea distribution and trophic ecology occurs in this species. We specifically wanted to answer a four-fold question: (1) Do female Cory’s shearwaters differ from males in their foraging strategies and distribution? Until now, most studies reported no sexual differences in the foraging behaviour of the species (e.g. refs 21–24, but see refs 19 and 20 for a counterpoint), especially during the chick-rearing period when the task of provisioning the growing chick is shared between sexes; (2) Will both sexes react differently to environmental stochasticity? The fact that females might be in poorer condition than males at the onset of the chick-rearing period, according to the ECH, should lead them to exploit pelagic habitats where prey distribution should be more predictable, based on conditions encountered in those areas during the pre-laying period^25^. Consequently, we expect females to show a more obvious shift in their foraging pattern in response to a decrease of marine productivity in the colony surroundings, when compared to males; (3) Will sexual segregation in foraging (e.g. habitat use) affect diet composition (inferred from stable isotopes) and if it does (4) which of the two, foraging strategies or dietary choices, leads to differential body condition between sexes? In this species, like for other marine predators, spatial segregation usually leads to isotopic segregation^26^, through the exploitation of different baseline isoscapes^25^, the choice of feeding on isotopically different prey species^23^ or a combination of both. Birds may increase their forging effort because they are in poorer body condition and need to restore their fat reserves^27^ or they can spend more time foraging and gain less weight just because food is less abundant^28^. Both situations may affect the overall breeding success^29^.

## Results

During 2010, 2011 and 2013 the North Atlantic Oscillation (NAO) index (Jun–Aug) was on average 5.7 units significantly lower, sea surface temperature (SST) within 200 km off Berlenga was 2.8 °C significantly higher, while the chlorophyll *a* concentration (CHL) was 0.89 mg m^−3^ significantly lower when compared to 2012, 2014 and 2015. Such inter-annual patterns on the environmental proxies of marine productivity in the colony surroundings seems to indicate that food availability in 2010, 2011 and 2013 was lower when compared to 2012, 2014 and 2015.

**Figure 1:**
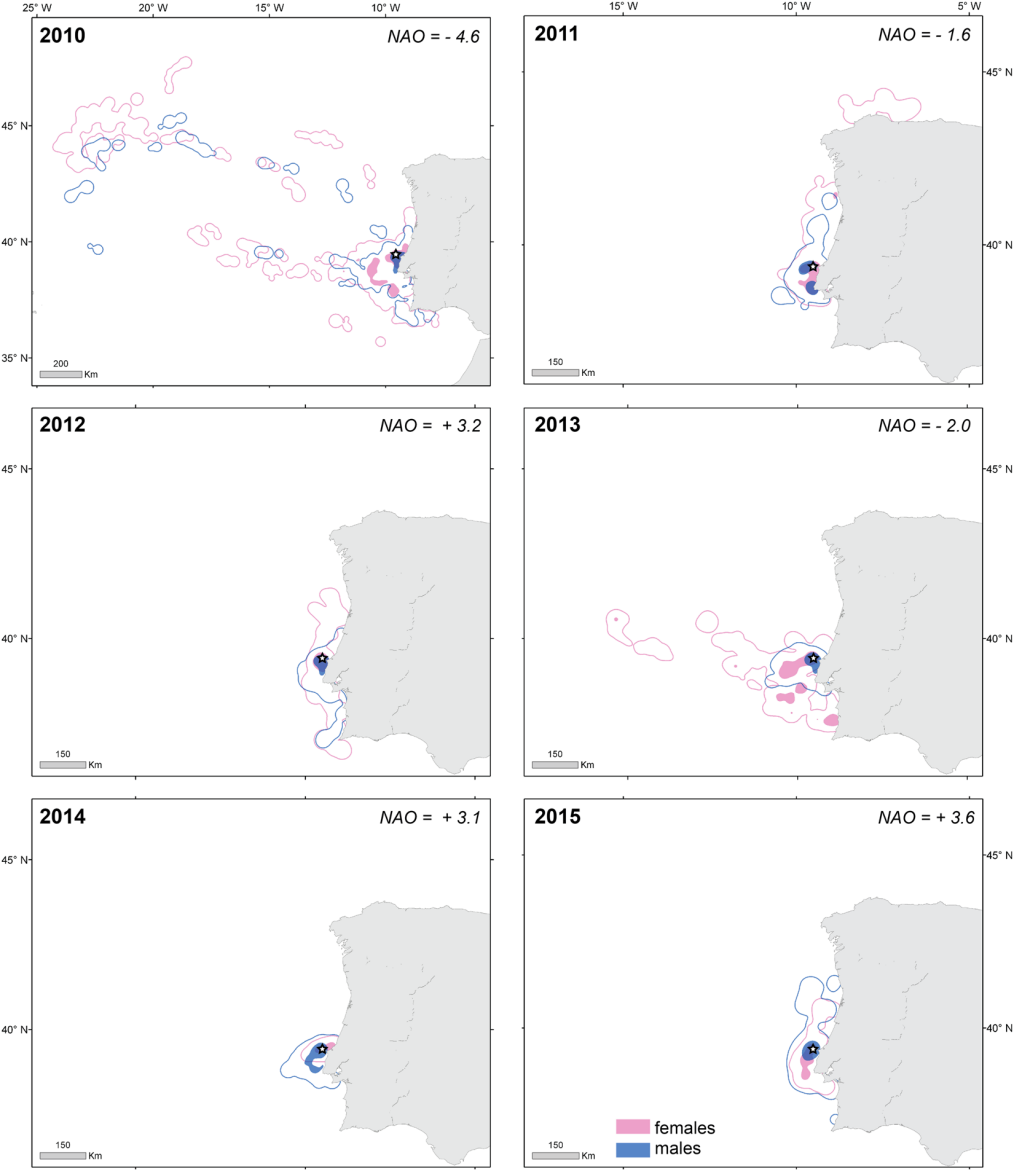
Home range (contour lines; 95% kernel UD) and foraging areas (filled areas; 50% kernel UD) of male (blue) and female (pink) Cory’s shearwaters during the chick-rearing seasons of 2010–2015. Kernel UD polygons generated with *adehabitatHR* package^44^ within the R environment version 3.2 (https://www.R-project.org/)^45^. Berlenga Island (breeding colony) represented with a white star. Also shown at the top-right corner of each map, the value of extended winter (December-March) North Atlantic Oscillation (NAO) index according to Hurrel 1995 (https://climatedataguide.ucar.edu/climate-data/hurrell-north-atlantic-oscillation-nao-index-station-based).

Also during 2010, 2011 and 2013, birds spent more 1.8 days during each foraging excursion, increased the long trips – short trips ratio by ~0.44 units, travelled ~187.3 km significantly further from their colony and spent more 14.1% of time in foraging areas when compared to 2012, 2014 and 2015. Moreover, females spent on average 1.0 days more in each foraging excursion, travelled ~101.8 km significantly further from their colony, and spent more 18.5% of time in area-restricted search (ARS) behaviour (i.e. foraging, not commuting) when compared to males (Tables 1 and 2, Fig. 1).

**Table 1:** Regional and local environmental predictors in the colony surroundings of female and male Cory’s shearwaters from Berlenga between 2010 and 2015; foraging trip characteristics, habitat of foraging areas, trophic ecology and body condition of both sexes.

	2010		2011		2012		2013		2014		2015	
	female	male	female	male	female	male	female	male	female	male	female	male
**Regional environmental predictors**												
Extended winter NAO index (Dec–Mar)	−4.6		−1.6		+3.2		−2.0		+3.1		+3.6	
NAO index (Jun – Sep)	−2.3 ± 0.5		−1.2 ± 0.3		+1.65 ± 0.2		−1.5 ± 0.4		+1.3 ± 0.4		+1.8 ± 0.7	
**Local environmental predictors (within 200 km)**												
Chlorophyll *a* concentration (CHL; mg m^−3^)	0.6 ± 0.2		1.7 ± 0.3		2.1 ± 0.1		1.3 ± 0.5		2.3 ± 0.3		2.0 ± 0.5	
Sea Surface Temperature (SST; °C)	21.9 ± 0.7		20.0 ± 1.5		17.2 ± 1.8		19.2 ± 1.4		16.9 ± 1.5		16.7 ± 0.8	
SST anomaly	1.8 ± 0.3		0.9 ± 0.2		−1.1 ± 0.4		0.7 ± 0.2		−0.9 ± 0.2		−0.7 ± 0.4	
**Foraging trip characteristics**												
N tracks [N birds]	69 [10]	50 [9]	19 [5]	21 [6]	34 [4]	40 [5]	22 [5]	20 [4]	15 [3]	16 [4]	30 [7]	63 [11]
Trip duration	3.9 ± 1.3	3.0 ± 1.0	3.2 ± 1.1	2.5 ± 1.0	1.5 ± 0.5	1.3 ± 0.5	2.8 ± 0.9	2.0 ± 1.2	1.4 ± 0.6	1.1 ± 0.5	1.5 ± 0.7	1.3 ± 0.6
Number of LT number of ST ^−1^	0.8 ± 0.6	0.5 ± 0.6	0.7 ± 0.5	0.4 ± 0.4	0.3 ± 0.1	0.2 ± 0.1	0.9 ± 0.6	0.5 ± 0.4	0.3 ± 0.2	0.4 ± 0.1	0.4 ± 0.1	0.2 ± 0.1
Maximum distance from colony (km)	823.2 ± 43.1	587.5 ± 54.4	624.2 ± 67.1	342.6 ± 67.1	194.4 ± 32.1	179.1 ± 44.0	542.9 ± 87.3	209.1 ± 66.9	98.1 ± 22.1	134.3 ± 22.2	58.4 ± 21.0	74.4 ± 21.9
Time spent flying trip^−1^ day^−1^(h)	8.6 ± 1.1	6.1 ± 1.0	6.6 ± 1.4	5.0 ± 1.3	4.9 ± 1.1	3.1 ± 1.6	6.8 ± 1.2	4.8 ± 1.0	4.3 ± 1.5	3.3 ± 1.9	5.4 ± 2.0	4.2 ± 0.9
% of time spent in foraging areas	45.2 ± 6.7	29.9 ± 5.5	37.5 ± 6.2	29.3 ± 5.0	18.9 ± 3.8	17.3 ± 3.7	38.3 ± 5.8	28.7 ± 5.6	18.0 ± 2.2	18.5 ± 4.4	16.3 ± 4.9	14.2 ± 3.5
**Habitat of foraging areas (within ARS zones)**												
Chlorophyll *a* concentration (CHL; mg m^−3^)	0.5 ± 0.3	1.1 ± 0.8	0.9 ± 0.4	1.4 ± 0.3	1.4 ± 0.5	1.7 ± 0.3	0.7 ± 0.5	1.3 ± 0.6	1.6 ± 0.7	1.9 ± 0.4	1.9 ± 0.9	2.1 ± 0.7
Sea Surface Temperature (SST; °C)	20.1 ± 0.7	18.3 ± 0.9	19.7 ± 0.9	18.0 ± 0.5	17.3 ± 0.7	17.1 ± 0.8	19.9 ± 0.6	18.6 ± 0.2	17.8 ± 0.4	17.9 ± 0.2	18.1 ± 0.4	17.8 ± 0.6
SST anomaly (ASST)	−0.7 ± 0.1	1.4 ± 0.6	−0.9 ± 0.6	1.1 ± 0.4	−2.1 ± 0.4	−1.8 ± 0.7	−0.8 ± 0.4	1.3 ± 0.5	−2.3 ± 0.5	−1.6 ± 0.9	−2.0 ± 0.7	−1.9 ± 1.1
**Trophic ecology**												
Plasma δ^13^C (‰)	−20.5 ± 0.5	−18.9 ± 0.7	−19.1 ± 0.6	−18.0 ± 0.4	−18.4 ± 0.6	−17.9 ± 0.5	−19.3 ± 0.4	−18.3 ± 0.5	−17.4 ± 0.2	−17.5 ± 0.3	−17.2 ± 0.6	−17.5 ± 0.3
Plasma δ^15^N (‰)	13.9 ± 0.4	14.4 ± 0.5	13.1 ± 0.4	14.0 ± 0.3	12.8 ± 0.3	13.0 ± 0.4	13.4 ± 0.3	14.1 ± 0.5	12.7 ± 0.4	13.5 ± 0.5	13.2 ± 0.3	13.9 ± 0.3
Plasma SEA_B_	1.8 ± 0.5	1.1 ± 0.6	1.3 ± 0.4	0.8 ± 0.3	0.8 ± 0.3	0.5 ± 0.2	1.2 ± 0.6	0.7 ± 0.2	0.7 ± 0.4	0.6 ± 0.3	0.9 ± 0.2	0.5 ± 0.2
**Body condition**												
Adults’ body condition index (BCI)	−1.2 ± 0.4	−0.5 ± 0.2	−0.9 ± 0.5	−0.5 ± 0.2	0.4 ± 0.5	0.7 ± 0.2	−0.8 ± 0.2	−0.4 ± 0.3	1.0 ± 0.2	1.2 ± 0.3	1.2 ± 0.3	1.4 ± 0.4
Mass gain trip duration^−1^ (g)	24.3 ± 8.3	29.0 ± 7.7	28.8 ± 9.3	31.0 ± 6.3	80.5 ± 9.9	81.6 ± 7.2	34.3 ± 8.3	40.9 ± 7.7	84.3 ± 6.3	86.9 ± 5.7	88.1 ± 5.6	90.0 ± 4.7

Extended winter (December-March) North Atlantic Oscillation (NAO) index according to Hurrell (https://climatedataguide.ucar.edu/climate-data/hurrell-north-atlantic-oscillation-nao-index-station-based). ARS – Area Restricted Search zones. LT – long trips (≥5 days of duration), ST – short trips (≤4 days of duration) as defined by ref. 24. Environmental predictors are for summer (June-September) of each year, unless otherwise stated. SEA_B_ **-** Bayesian approximation of the standard ellipse area (see Jackson *et al*. 2011 for more details on these metrics of isotopic niche width). Values are mean ± SD.

**Table 2:** Generalized Linear Mixed Models (GLMMs) testing the effect of the interaction between year (2010–2015) and sex (male and female) on regional and local environmental predictors in the colony surroundings (200 km around the breeding colony), foraging habitat, trip characteristics, spatial ecology and body condition shown in Table 1.

Variables	Year			Sex			Year*Sex		
	GLMM	P	Effect	GLMM	P	Effect	GLMM	P	Effect
**Regional environmental predictors**									
Extended winter NAO index (December - March)	—	—	—	—	—	—	—	—	—
NAO index (Jun - Aug)	F_4,15_ = 29.36	**<0.001**	10,11,13 < 12,14,15	—	—	—	—	—	—
**Local environmental predictors (within 60 km)**						—			
Chlorophyll *a* concentration (CHL; mg m^−3^)	F_4,167_ = 2.42	0.11	—	—	—	—	—	—	—
Sea Surface Temperature (SST; °C)	F_4,167_ = 3.45	**0.01**	10,11,13 > 12,14,15	—	—	—	—	—	—
**Foraging trip characteristics**									
N tracks [N birds]	—	—	—	—	—	—	—	—	—
Trip duration (d)	F_11,337_ = 1.89	**0.04**	10,11,13 > 12,14,15	F_11,337_ = 2.10	**0.02**	females > males	F_11,337_ = 1.62	0.09	—
Number of LT number of ST ^−1^	F_11,337_ = 2.33	**0.01**	10,11,13 > 12,14,15	F_11,337_ = 2.36	**0.01**	females > males	F_11,337_ = 1.99	**0.03**	10, 11,13 females > all others
Maximum distance from colony (km)	F_11,337_ = 2.11	**0.03**	10,11,13 > 12,14,15	F_11,337_ = 3.78	**0.001**	females > males	F_11,337_ = 2.09	**0.04**	10 females > all others
Time spent flying trip^−1^ day^−1^(h)	F_11,337_ = 1.51	0.15	—	F_11,337_ = 1.22	0.21	—	—	—	—
% of time spent in foraging areas	F_11,337_ = 10.02	**<0.001**	10,11,13 > 12,14,15	F_11,337_ = 2.00	**0.04**	females > males	F_11,337_ = 1.38	0.19	—
**Habitat of foraging areas (within ARS zones)**									
Chlorophyll *a* concentration (CHL; mg m^−3^)	F_11,337_ = 1.98	**0.04**	10 < all other years	—	—	—	—	—	—
Sea Surface Temperature (SST; °C)	F_11,337_ = 3.24	**0.001**	10, 11,13 > 12,14,15	F_11,337_ = 1.65	0.11	—	F_11,337_ = 1.37	0.21	—
SST anomaly (ASST)	F_11,337_ = 2.48	**0.01**	10, 11,13 > 2012,14,15	F_11,337_ = 3.23	**0.001**	females < males	F_11,337_ = 2.22	**0.02**	10, 11,13 females<all others
**Trophic ecology**									
Plasma δ^13^C (‰)	F_11,337_ = 2.11	**0.03**	10,11,13 < 12,14,15	F_11,337_ = 1.59	0.13	—	F_11,337_ = 1.68	0.11	—
Plasma δ^15^N (‰)	F_11,337_ = 2.47	**0.01**	10,11,13 12,14,15	F_11,337_ = 3.32	**0.001**	females < males	F_11,337_ = 2.01	**0.04**	10, 11,13 females < all others
Plasma SEA_B_	F_11,337_ = 1.48	0.17	—	F_11,337_ = 1.92	**0.05**	females > males	F_11,337_ = 1.40	0.19	
**Body condition**									
Adults’ body condition index (BCI)	F_11,337_ = 2.48	**0.01**	10,11,13 < 12,14,15	F_11,337_ = 3.25	**0.001**	females < males	F_11,337_ = 2.55	**0.01**	10 females < all others
Mass gain trip duration ^−1^ (g)	F_11,337_ = 2.07	**0.02**	10,11,13 < 12,14,15	F_11,337_ = 1.35	0.18	—			

ARS – Area Restricted Search. LT – long trips (≥5 days of duration), ST – short trips (≤4 days of duration) as defined by ref. 24. Study years represented by the last two digits. The individual was used as a random effect to avoid pseudo-replication issues. Significant results in bold. Effect was evaluated with Post-hoc multiple comparisons with Bonferroni correction.

SST and SST anomaly (ASST) were on average 3.2 and 4.1 °C significantly higher, respectively, in 2010, 2011 and 2013 than in 2012, 2014 and 2015, while CHL was 1.1 mgm^−3^ lower in 2010 when compared to the other five years. The plasma δ^13^C and δ^15^N values were 2.1 and 1.5‰ significantly lower and higher, respectively, in 2010, 2011, 2014 than in 2012, 2013 and 2015. Females showed a plasma δ^15^N value 2.1‰ lower and a plasma Bayesian estimate of the standard ellipse (SEA_B_) 1.2 units higher than males. Body condition index (BCI) of adults was 55% significantly lower and mass gain per trip duration (i.e. mass gain when returning from a foraging trip divided by the number of days spent at sea) was 62% lower in 2010, 2011, 2013 than in 2012, 2014 and 2015, while females were in significantly poorer body condition (40% lower) and generally gained less mass per foraging excursion (37% less) when compared to males (Tables 1 and 2).

During 2010, 2011 and 2013 there was a significantly smaller overlap between sexes for the 50%, 95% and 99% Kernel UD, compared to 2012, 2014 and 2015, with a mean increase by ~26% in the overlap of the entire UDs (Table 3).

**Table 3:** Observed and randomized overlap between female and male Cory’s shearwaters Kernel Utilization Distributions (Kernel UD).

Kernel UD	Year	Observed	Randomized	*P*
50%	2010	0.34	1.29	<**0.001**
	2011	0.32	1.31	<**0.001**
	2012	0.44	0.48	0.12
	2013	0.31	0.92	**0.001**
	2014	0.45	0.49	0.15
	2015	0.49	1.11	0.20
95%	2010	0.56	1.05	**0.001**
	2011	0.53	0.98	**0.01**
	2012	0.68	0.73	0.11
	2013	0.51	1.07	**0.001**
	2014	0.66	0.81	0.19
	2015	0.70	0.87	0.21
99%	2010	0.61	1.07	**0.01**
	2011	0.55	1.01	**0.02**
	2012	0.71	0.74	0.19
	2013	0.52	1.11	**0.01**
	2014	0.69	0.85	0.11
	2015	0.72	0.88	0.19

*P* represents the proportion of randomized overlaps that were smaller than the observed overlap. Significant differences are shown in bold.

**Figure 2:**
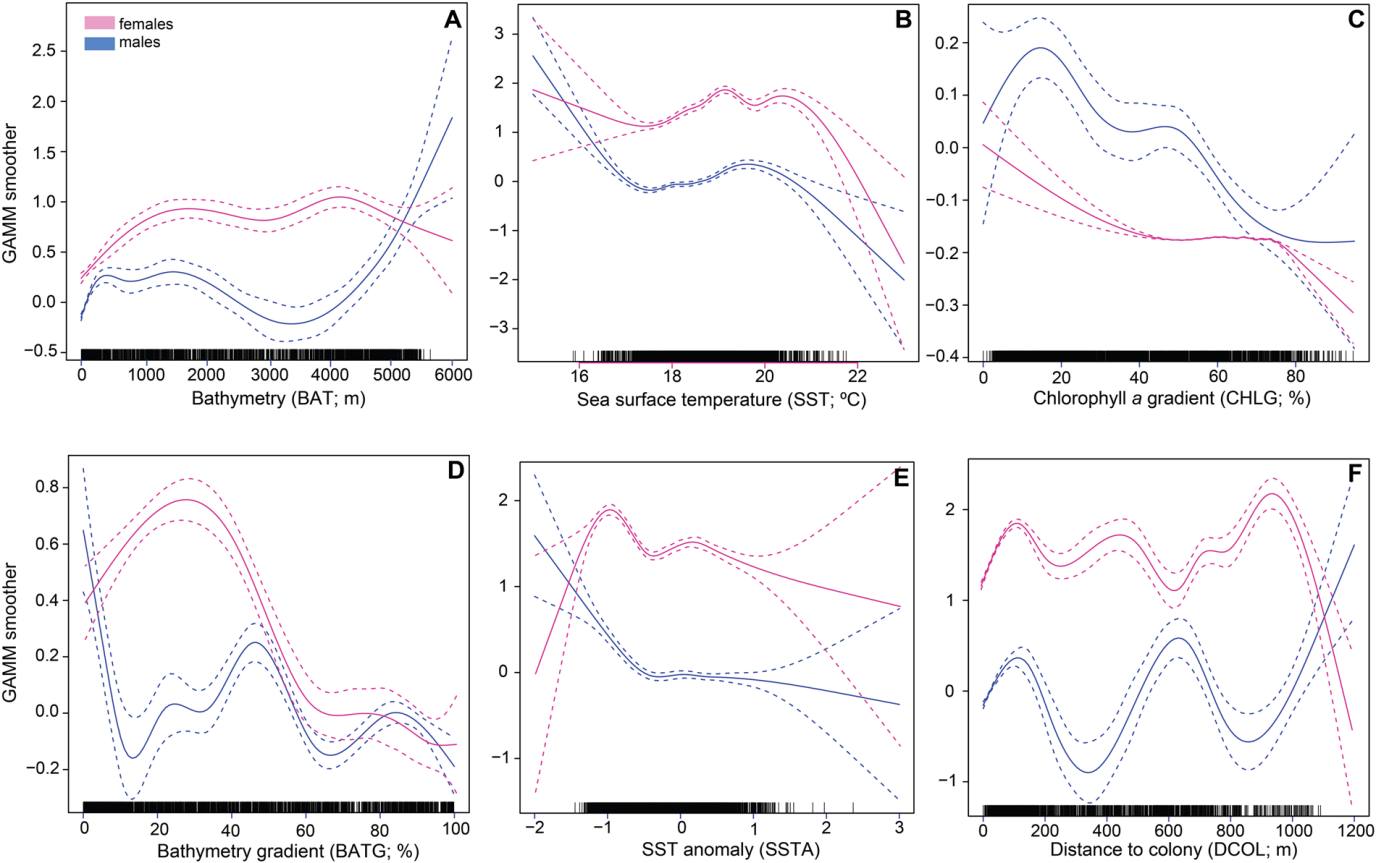
Response curves of the most important variables explaining the foraging distribution of male (blue) and female (pink) Cory’s shearwaters. Habitat selection functions for (**A**) bathymetry, (**B**) sea surface temperature, (**C**) chlorophyll *a* gradient, (**D**) bathymetry gradient, (**E**) sea surface temperature anomaly and (**F**) distance to land. Plots show the predicted curve from the best models (solid lines) and 95% confidence intervals (dashed lines) for male (blue) and female (pink) Cory’s shearwaters. GAMM – Generalized Additive Mixed Model.

Overall, generalized additive mixed models (GAMMs) showed a good predictable capacity, explaining >22.4% and >22.7% of the deviance in the first-passage time (FPT) duration (proxy of foraging activity) of females and males respectively (according to Table 4). In females, FPT duration increased with increasing bathymetry (BAT) and BAT gradient (BATG) and decreasing SST and distance to colony (DCOL) (Fig. 2). In males, FPT duration increased with increasing BAT, BATG and CHL gradient (CHLG) and decreasing SST and DCOL (Table 4, Fig. 2).

**Table 4:** Parameter estimates (±SE) from Generalized Additive Mixed Models (GAMMs) fitted to the Area Restricted Search behaviour (i.e. First Passage Time – FPT – duration; proxy of foraging activity) of female and male Cory’s shearwaters showing the ranking on the candidate models based on the corrected Akaike Information Criteria (AICc).

Model structure	FPT duration in females			ED	Model structure	FPT duration in males			ED
	AICc	ΔAICc	AICc Wgt			AICc	ΔAICc	AICc Wgt	
BAT * BATG + DCOL	243.8	0.01	0.79	42.1	BAT * BATG	162.3	0.07	0.64	39.2
SST * SSTA * Year	302.0	1.45	0.62	35.2	SST + DCOL	199.2	1.01	0.52	30.0
Constant	602.9	2.15	0.11	9.1	CHLG	277.0	1.55	0.44	22.7
					DCOL * Year	309.7	1.76	0.39	31.2
					Constant	528.4	2.46	0.12	10.9
**Fixed effects**	***β*** ± **SE**	**z**	**P**		**Fixed effects**	***β*** ± **SE**	**z**	**P**	
Intercept	1.35 ± 0.07	19.45	**<0.001**		Intercept	−0.94 ± 0.19	−18.84	**<0.001**	
BAT: BATG	4.23 ± 0.22	15.23	**<0.001**		BAT: BATG	7.12 ± 0.14	23.12	**<0.001**	
DCOL	−1.23 ± 0.09	3.15	**0.10**		SST	−4.14 ± 0.23	−16.23	**<0.001**	
SST: SSTA: Year	−2.65 ± 0.14	−9.24	**0.001**		DCOL	−2.36 ± 0.11	−7.12	**0.01**	
					CHLG	5.24 ± 0.21	18.23	**<0.001**	
					DCOL: Year	4.12 ± 0.25	14.12	**0.001**	
Random intercept for Bird_ID (variance ± SD)	0.10 ± 0.02				Random intercept for Bird_ID (variance ± SD)	0.15 ± 0.09			

All evaluated models included individual identity as a random factor. Models are ordered by the AIC value. Presented are the habitat variables in the top-ranked models (ΔAICc < 2). ED – Explained deviance (%); BAT – Bathymetry (m); BATG –BAT gradient (%); SST - Sea Surface Temperature (°C); SSTG –SST gradient (%); CHL – Chlorophyll *a* concentration (mgm^−3^); CHLG – CHL gradient; DCOL – Distance to colony (m). Year – 2010–2015. Significant results in bold.

## Discussion

Our study documented a clear sexual segregation in foraging by Cory’s shearwaters in years of increased environmental stochasticity, i.e. likely years of low food availability. During years of good environmental conditions, both sexes foraged mostly within the colony surroundings, although females tended to exploit in more pelagic waters. In years of greater stochasticity, females undertook much longer foraging trips, similar to those performed during the pre-laying^25^ and incubation^30^ periods, which decreased their body condition. They also enlarged their isotopic niche, but fed mostly on lower trophic level prey, when compared to males. Conversely, males kept foraging in the colony surroundings, maybe outcompeting females to access comparatively higher trophic level preys, which translated into a higher body condition index and daily mass gain during foraging trips.

### Environmental factors driving sexual segregation in foraging

Negative NAO index values for 2010, 2011 and 2013 concurred with significant increases in SST and reductions in CHL, contrasting to what happened in 2012, 2014 and 2015, when positive NAO values where observed^28^. Even though at-sea patterns were generally similar between sexes, sexual segregation was observed in years of greater environmental stochasticity, when marine productivity was lowest. Birds travelled greater distances from their colony, and spent more time in foraging areas than they did during years of higher marine productivity. Therefore, previous studies finding no evidence of sexual segregation in foraging strategies of breeding Cory’s shearwater^20, 31^ are partly explained by the fact that they were conducted solely during the incubation period, and presumably during years of relatively good environmental conditions. In fact, Ramos *et al*.^20^ suggested that more subtle sexual differences might exist, for instance in diving behaviour, or be detected in resource partitioning (e.g., sexual differences in the size of consumed prey). While we did not examine such factors in this study, we could show significant sexual differences in foraging distribution and oceanographic features within the birds’ foraging areas, as well as in several aspects of their trophic ecology. Foraging trip characteristics, features of the foraging areas (e.g. SST) and body condition of females during years of poor environmental conditions were significantly affected, and thus differed from those of males, (and also from females during years of good environmental conditions). Therefore, our study suggests that sexual differences in the foraging ecology of Cory’s shearwaters are likely to be perceptible under poor environmental conditions. Our results make sense in the light of the competitive exclusion by the dominant sex, i.e. males over females^3^, which will occur mostly when environmental conditions are poor and resources are scarce. Furthermore, previous studies regarding such effects were conducted mostly during a single breeding season, and thus failed to detect any important influence of environmental conditions on sexual segregation in foraging (e.g. ref. 22).

### Differential effects of foraging choices on the trophic ecology of both sexes

Overall, the carbon isotopic values suggest that males used to feed on prey from coastal environments (i.e. higher δ^13^C value) when compared to females, which exhibited a more pelagic foraging behaviour^32^. While foraging over more pelagic environments, mostly during 2010, 2011 and 2013, females showed lower δ^15^N values, which suggests they were feeding on lower trophic level prey than males. There might be at least two reasons for this; 1) females were more explorative in 2010, 2011 and 2013 (i.e. years of poor environmental conditions, likely with lower food availability), feeding on prey of comparatively lower δ^15^N values, such as small pelagic fish species (e.g. *Scomber sp*; ref. 23) and enlarging their isotopic niche or, 2) in years of food scarcity birds tend to attend more to fishery discards^33^ where males might outcompete females for offal and discards and gain access to higher trophic level prey. This has been reported to occur in several Procellariform species (e.g. northern giant petrels *Macronectes halli* ^34^), potentially leading to sex-biased bycatch rates (see review by Gianuca *et al*.^35^). Even though Cory’s shearwaters are considered to interact much less with fisheries and/or to be much less frequently victim of by-catch than their Mediterranean congeneric, they have been observed feeding on fishery discards^36, 37^. Although both hypotheses can be true, only the collection of more tracking and blood data along with vessel monitoring system data during subsequent years will allow disentangling the major driver of this sexual isotopic segregation pattern. Nevertheless, both groups were isotopically segregated even in years where both sexes were foraging in similar regions (i.e., a higher foraging area overlap in 2012, 2014 and 2015 than during the other study years), which certainly results from individuals feeding on isotopically different prey^38^.

### Final remarks

Our results are compatible with the interpretation that sexual segregation in Cory’s shearwaters might be mediated by habitat segregation from neritic to oceanic regimes (by males and females, respectively), with such spatial segregation occurring mostly in years of increased environmental stochasticity. Multi-year tracking studies are thus crucial (6 breeding seasons in our study) to successfully detect even relatively small sexual differences in the foraging ecology and behaviour of marine predators. Such small differences may be ecologically relevant because sexual competition for trophic resources may be stronger when environmental conditions are poor. A greater foraging success of one sex over the other may result in differential body condition of pair mates to endure on reproductive duties, thus resulting in an increased probability of breeding failure.

## Methods

### Fieldwork

Seventy-three Cory’s shearwaters (N = 34 females and N = 39 males) were tracked during several foraging trips (N = 139 female and N = 210 male) with GPS-loggers (IgotU GT-120; Mobile Action Technology Inc., Taiwan) on Berlenga Island located in the Portuguese coast (39°23′N, 9°36′W), during 15-August – 15 September (mid chick-rearing period) of 2010–2015 (Table 1). The devices weighed 17 g, which represented between 2.2% and 2.9% (median = 2.6%) of the birds’ mass and was below the 3% threshold advised by ref. 39. Devices were set to record locations each 5 minutes. GPS loggers were then attached using TESA® tape to the contour feathers along and in between both scapulas. The whole process took less than 10 minutes, thus minimizing the overall stress to the animal. Upon logger retrieval, a blood sample of about 0.5 ml was collected from the tarsal vein of each individual for stable isotope analysis (SIA). The tracked birds were measured (gonys height and the length of wing, tarsus and culmen) and weighed at both capture and recapture. Body measurements (except body mass) were included on a Principal Component Analysis (PCA) and the PC1 scores were used as a measure of structural body size to compute a body condition index (BCI). This index was obtained from the residuals of the linear regression of body mass on PC 1 scores. We used BCI as an indicator of energetic reserves (i.e. fitness parameter).

All animals were handled in strict accordance with good animal practice as defined by the current European legislation. All animal work was approved by the Portuguese Government (ICNF) under licenses: 188/2010/CAPT, 152/2011/CAPT, 101/2012/CAPT, 99/2013/CAPT, 203/2014/CAPT, 169/2015/CAPT.

### Area - Restricted Search (ARS) zones

Fauchald and Tveraa^40^ developed a technique, named first passage time (FPT) to assess the spatial scale that animals use to encounter their prey. FPT is, by definition, the time required for an animal to pass through a circle with a given radius *r*. By moving this circle along the path of the animal, we will obtain a scale-dependent measure of search effort and therefore the behavioural response of an individual in the environment. Because top marine predators usually forage in a patchy and hierarchical environment^41^, increases in the turning rate and/or decreases in speed of its foraging path should be related to the so-called area-restricted search (ARS) behaviour. ARS will then appear as an individual reaction to changes in resource availability and distribution, by increasing the residence time in the productive patch^40^.

Zones of area-restricted search (ARS) were estimated applying FPT analysis, following^40^ and using software R 3.0 (R Development Core Team 2014). Locations were first projected onto a Lambert equal-area projection. Usually, in water positions result in very small-scale ARS zones (<100 m diameter), which considerably increases the variance in FPT and can camouflage larger-scale ARS zone^42^. To address this problem, we removed bouts on the water and interpolated locations to obtain a distance interval of 0.1 km for FPT analysis^43^. We considered positions with speed <3 km as resting or preening behaviours on the water or inland, after inspecting the frequency distribution of speeds. Following the recommendations of ref. 43, FPT analysis was performed in two steps: (1) to detect large-scale ARS we ran the analysis on the whole path, estimating the FPT every 1 km for a radius *r* from 1 to 50 km; (2) to detect small spatial scale events we run again FPT analysis every 0.1 km for an *r* varying between 0.1 and 10 km. The plot representing variance in log (FPT) as a function of *r* allowed us to identify the ARS scales by peaks in the variance. In this calculation, FPT was log transformed to make the variance independent of the magnitude of the mean FPT^40^. It is also possible to locate where the bird entered an ARS zone and the time spent in this area by plotting FPT values where a peak of variance occurred as a function of time since departure from the colony. ARS locations were also used to feed the Generalized Additive Mixed Models (GAMMs).

### Habitat use

GPS locations of each bird where ARS behaviour was detected (ARS zones) were examined under the *adehabitatHR* R package^44^ generating Kernel Utilization Distribution (Kernel UD) estimates within the R environment^45^. The most appropriate smoothing parameter (*h*) was chosen via least squares cross–validation for the unsmoothed GPS data, and then applied as standard for the other datasets and grid size was set at 0.05° (to match the grid of environmental predictors). We considered the 50% and 95% kernel UD contours to represent the core foraging areas (FR) and the home range (HR), respectively.

The extent of within-year overlap between male and female home ranges was estimated using using a kernel UD overlap index, which is considered the most appropriate measure of overlapping space use^46^. We used a randomization technique (1000 randomizations of our dataset) to test the null hypothesis that there was no difference in the spatial distribution of males and females in each study year. If the null hypothesis is true, overlap between males and female 50% and 95% kernel UDs should not differ significantly from that calculated if sex were randomly assigned. *P*-values were determined by the proportion of random overlaps that were smaller than the observed overlap (see refs 18, 47 and 48 for a similar approaches). More details on measures of spatial overlap can be found in ref. 46.

### Environmental data

The extended winter North Atlantic Oscillation (NAO) index was used as a large-scale environmental predictor for the North Atlantic area, and specifically for the Western Iberia Upwelling Ecosystem (WIUE). The NAO index refers to a north–south alternation in atmospheric mass between the subtropical Atlantic and the Arctic, and thus involves out-of-phase behaviour between the climatological low-pressure centre near Iceland and the high-pressure centre near the Azores. In other words, the index is the result of the difference of normalised sea-level pressures (Pa) between the former two locations (https://climatedataguide.ucar.edu/climate-data/hurrell-north-atlantic-oscillation-nao-index-station-based). In general, a low NAO index value would be depicting an intense upwelling and low SST due to stronger winds (during the previous winter). Under these conditions, we should expect a lower abundance of plankton^49^ and consequently lower abundance and availability of fish prey to top marine predators, such as seabirds. In some upwelling regions, such as the Portuguese coast, an overly intense upwelling may be responsible for a very low recruitment of small pelagic fish (such as sardine *Sardina* spp.) and low abundance of plankton because, under these conditions, the fish larvae and plankton are driven offshore, which consequently increases larvae mortality and creates a spatial mismatch of plankton and juvenile planktonic fishes a few months later^50^. In addition, the effect of the NAO is regionally dependent, and a reverse effect (i.e. weaker wind fields and higher SST) should occur in the northern and western Atlantic regions^51^.

We used small-scale environmental predictors, such as chlorophyll *a* concentration (CHL) and sea surface temperature (SST) data, downloaded from http://oceanocolor.gsfc.nasa.gov/, as daily night-time products with a resolution of 0.04° (approx. 4 km) in the SMI-HDF format. Bathymetric data (BAT), taken as water depth, was downloaded from the ETOPO2v2 database at a spatial resolution of 0.03° (approximately 3 km; http://www.ngdc.noaa.gov/mgg/fliers/01mgg04.html). HDF files were converted to raster using the Marine Geospatial Ecology Tools in ArcGIS v10.1^52^, and then to ASCII to create composites. All composites were constructed using the freeware R environment and different functions of the *raster* package. Spatial gradients of SST, CHL and BAT (SSTG, CHLG and BATG, respectively) were obtained by estimating the proportional change (PC) within a surrounding 3 × 3 cell grid using a moving window as follows: PC = [(maximum value − minimum value) × 100/maximum value]^53^. SSTG and CHLG are believed to be good indicators of oceanic fronts, while the BATG was used as a proxy for slope. Additionally, two static variables were generated. Distance to colony (DCOL) was calculated using the *distance* tool (*spatial analyst* toolbox) in ArcGIS v10.1.

### Stable isotope analysis (SIA)

Stable-nitrogen isotope ratios (^15^N: ^14^N, expressed as δ^15^N) and stable- carbon isotope ratios (^13^C: ^12^C, expressed as δ^13^C) in the plasma of Cory’s shearwater were determined to investigate the trophic choices of each sex during each year. Plasma has a half-life of about 3.5 days^54^ (i.e. high turnover rate), therefore it represents prey ingestion and trophic ecology of tracked individuals during the last trips before sampling^55^. The δ^15^N is mainly used to define the trophic position of the consumer^56^, while δ^13^C reflects the foraging habitat of the consumer^57^. There is a gradient of high to low values of δ^13^C from benthic and inshore to pelagic and offshore food webs, because the organic enrichment at the coast is gradually diluted towards the open ocean^58^.

Each of the tracked birds was sampled upon return from a foraging trip. Blood samples (around 0.5 ml) were collected from the tarsal or brachial vein using insulin-syringes with 27 G needles. Blood samples were then separated into plasma and RBC by centrifugation at 12000 rpm for 5 min, within 2–4 hours of sampling and stored frozen at −20 °C until preparation for analysis. Successive rinses with a 2:1 chloroform-methanol solution were performed on the plasma for delipidation^55^. Isotope ratios of carbon and nitrogen of plasma were then determined by continuous-flow isotope ratio mass spectrometry, using an EA-IRMS (Isoprime, Micromass, UK). The analytical precision for the measurement was 0.2‰ for both carbon and nitrogen. All values presented are means ± 1 SD unless otherwise stated.

### Statistical analysis

Generalized Linear Mixed Models (GLMMs) tested the effect of (1) year (2010–2015), (2) sex and, (3) the interaction between year and sex (i.e. independent variables) on the mean values of (1) NAO index (Jun–Sep), (2) CHL, SST and SST anomaly (within 200 km of the breeding colony), (3) trip duration, number of long trips number of short trips^−1^ (with long trips, ≥5 days of duration and short trips, ≤4 days of duration as defined by ref. 24), max. distance from colony, time spent flying trip^−1^ day^−1^, % of time spent in foraging areas (ARS zones), (4) CHL, SST and SST anomaly within ARS zones, (5) δ^13^C, δ^15^N and SEA_B_ of plasma and (6) adults’ body condition index (BCI) and mass gain trip duration^−1^ (i.e. dependent variables). Trip identity was nested within the individual as a random term to avoid potential pseudo-replication problems, since all individual birds performed multiple trips. Gaussian distribution of error terms and a log-link function were used in the modelling. Post-hoc multiple comparisons with Bonferroni correction were used to identify significant differences between categories of each independent variable. R packages used in the GLMMs were *lme4* ^59^ and *lmerTest* ^60^.

When modelling the occurrence of ARS behaviour (First Passage Time – FPT – duration) in male and female Cory’s shearwaters we used GAMMs to (1) select the most parsimonious models explaining FPT and (2) estimate smoothers for each of the environmental parameters for the top-ranked models (ΔAICc < 2). GAMMs combine the utilities of linear mixed models^61^ and generalized additive models^62^ so that random factors, fixed factors and nonlinear predictor variables can all be estimated in the same statistical model. Separate models were developed for male and female birds aiming at simpler interpretations of their outputs (i.e. interactions with the variable sex would be difficult to interpret in complex models). Such models included (1) year and (2) all different environmental predictors of productivity (e.g. SST) as fixed factors, trip identity within bird identity as a random term (to account for pseudoreplication issues).

As part of the GAM functions within the *mgcv* R package ref. 63 the smoothing parameter is chosen automatically using generalised cross-validation (GCV). In order to model spatial auto-correlation we included an isotropic thin plate spline which is set up as a two-dimensional smoother based on both x and y coordinates (i.e. *s*(x,y)). Incorporating a spatial smoother is one means of modelling a spatial trend within a model, more details on this approach can be found in ref. 63. Prior to modelling we examined the correlations between all environmental variables in order to ascertain whether collinearity may have occurred. We assumed that a Spearman correlation coefficient higher than 0.5 was problematic, and thus the environmental predictor (from the pair of highly correlated ones) which produced the highest Akaike information criteria (AIC) value on a univariate analysis was excluded. Initially we restricted GAMMs to a maximum of 5 knots to prevent over-fitting, however if GAMMs failed diagnostic checks we increased the number of knots until these checks were satisfactory. For the spatial smoothers in the models we used the default settings in the *mgcv* package^63^ to estimate the number of knots required. When performing GAMMs, minimum adequate models were selected by backwards selection, using *K*-fold cross-validation, following^18, 62^.

To establish the isotopic niche among periods with the stable isotope data we applied the recent metric SIBER (Stable Isotope Bayesian Ellipses in R), which is based on a Bayesian framework that confers a robust comparison to be made among data sets concerning different sample sizes^64^. The area of the standard ellipse (SEAc, an ellipse having a 40% probability of containing a subsequently sampled datum) was adopted to compare female and male isotopic values and their overlap in relation to the total niche width (both groups combined), and a Bayesian estimate of the standard ellipse and its area (SEA_B_) was used to test whether females’ isotopic niche is narrower than males’ isotopic niche (i.e. *p*, the proportion of ellipses in female birds that were smaller than in male individuals; see ref. 64 for more details). All the metrics were calculated using standard.ellipse and *convexhull* functions from SIBER implemented in the package SIAR (Stable Isotope Analysis in R^65^); under R^45^. All data are presented as mean ± SD, unless otherwise stated. Results were considered significant at P ≤ 0.05.
